# Integrated Metabolomic and Microbiome Profiling Reveals Divergent Effects of No- and High-Fat Coffee in Mice

**DOI:** 10.3390/nu18121939

**Published:** 2026-06-16

**Authors:** Xinye Gong, Yuelin Wang, Wenbo Chu, Yijie Zhao, Jia Liu, Qinghua Zou

**Affiliations:** 1 Department of Microbiology, School of Basic Medical Sciences, Peking University Health Science Center, Beijing 100191, China; 2 Department of Laboratory Animal Science, Peking University Health Science Center, Beijing 100191, China

**Keywords:** coffee, high-fat coffee, metabolomics, 16S rRNA sequencing, gut microbiota

## Abstract

**Background/Objectives** Coffee is widely consumed worldwide and is rich in bioactive compounds with potential metabolic benefits. Recently, lipid-enriched coffee formulations have gained popularity; however, their biological effects and underlying mechanisms remain poorly understood. **Methods** In this study, we employed an integrated multi-omics approach to investigate the impact of coffee and high-fat coffee on the plasma metabolome and gut microbiota of C57BL/6J mice. Eighteen male mice were randomly assigned to three groups (*n* = 6) and received water, coffee, or high-fat coffee by oral gavage daily for 14 days. The plasma metabolome was analyzed via UHPLC-MS/MS, and the gut microbiota was profiled u 16S rRNA gene sequencing. **Results** Metabolomic analysis revealed distinct clustering patterns among groups. A total of 200 metabolites were significantly altered in the coffee group compared with the water group, while 86 metabolites were altered in the high-fat coffee group compared with the coffee group, with 56 overlapping metabolites suggesting a core metabolic response. Microbiome analysis showed that coffee consumption increased the abundances of *Akkermansia* and *Bifidobacterium* and decreased the levels of *Ligilactobacillus* and *Muribaculum*. Coriobacteriaceae UCG-002 and *Turicibacter* were significantly enriched in the high-fat coffee group, whereas Lachnospiraceae NK4A136 group, *Mucispirillum* and unclassified Lactobacillaceae were reduced. Association analysis highlighted the top 20 metabolites with the highest degree of connection to gut microbial genera, two of which belong to the chlorogenic acid pathway. **Conclusions** Reduced levels of ferulic acid and 3-hydroxybenzoic acid, two metabolites potentially involved in antioxidant and anti-inflammatory activities, were observed in the high-fat coffee group, suggesting that dietary cream influences microbiota-associated chlorogenic acid metabolism.

## 1. Introduction

Coffee is one of the most widely consumed beverages worldwide and has a complex dietary exposure rich in bioactive compounds, including caffeine, chlorogenic acids, diterpenes, and polyphenols, which have been extensively associated with diverse physiological effects on host metabolism [1]. Accumulating epidemiological and experimental evidence suggests that habitual coffee consumption is linked to improved metabolic health, including reduced risks of obesity [2], type 2 diabetes [3], and metabolic syndrome [4], potentially through modulation of energy homeostasis, glucose metabolism, and inflammatory pathways. Chlorogenic acids, the most abundant polyphenols in coffee, exhibit low intestinal absorption and their bioavailability largely depends on metabolism by the gut microbiota, yielding microbial metabolites that contribute significantly to their biological activities [5]. In recent years, however, the emergence of lipid-enriched coffee formulations—commonly referred to as high-fat coffee, often prepared by adding butter, cream, or medium-chain triglyceride (MCT) oil—has gained increasing popularity due to claims of enhanced energy supply, satiety, and cognitive performance. Unlike traditional coffee, high-fat coffee introduces a substantial lipid load that may profoundly influence host metabolic processes, including lipid absorption, ketogenesis, and insulin sensitivity. Despite its growing consumption, the biological effects of high-fat coffee remain insufficiently characterized, particularly in comparison to conventional coffee. Li Chen et al. [6] employed 16S rRNA sequencing and serum metabolomics to investigate the effects of caffeine on high-fat diet-induced obese mice, demonstrating that caffeine supplementation significantly reshaped gut microbial composition and altered circulating metabolites, ultimately ameliorating metabolic syndrome. However, it is still unclear how ingestion of coffee in high-fat formulations influences plasma metabolic profiles and gut microbial structure.

Metabolomics has emerged as a powerful analytical approach for the comprehensive characterization of small-molecule metabolites in biological systems [7], providing a direct functional readout of physiological and pathological states. By capturing dynamic changes in endogenous and exogenous metabolites, metabolomics enables the systematic investigation of metabolic pathways and their responses to dietary interventions, environmental factors, and disease conditions.

Parallel to metabolomics, 16S rRNA gene sequencing has become a cornerstone technique for profiling the composition and diversity of microbial communities, particularly within the gut microbiota [8]. This method relies on the amplification and sequencing of conserved regions of the bacterial 16S ribosomal RNA gene interspersed with hypervariable regions, which enable taxonomic classification across different phylogenetic levels. With the rapid development of next-generation sequencing platforms and bioinformatic pipelines, 16S rRNA sequencing allows high-throughput, cost-effective, and relatively accurate characterization of microbial community structures in complex samples. Given the critical role of the gut microbiota in regulating host metabolism, immune function, and energy balance, 16S rRNA-based microbial profiling serves as an essential tool for exploring host–microbiota interactions and their contributions to metabolic phenotypes.

In this study, we employed an integrated multi-omics approach to systematically investigate the effects of coffee and high-fat coffee consumption on host metabolism and gut microbial communities using C57BL/6J mice, a strain chosen for its well-characterized genetic background and physiological similarity to humans. We focused on cream-based high-fat coffee, where dairy cream served as the primary lipid source, closely reflecting widely consumed commercial products such as lattes or cream-added coffee. We aim to characterize the metabolic and microbial responses to coffee and high-fat coffee consumption, with a particular focus on whether the addition of dietary lipids influences the bioavailability or microbial conversion of coffee-derived bioactive compounds, which thereby affects the systemic metabolic effects of coffee.

## 2. Experimental Section

### 2.1. Coffee, Mice and Experimental Design

Eighteen healthy five-week-old male C57BL/6J mice were obtained from the Department of Laboratory Animal Science of Peking University Health Science Center. Only male mice were used to avoid estrous-cycle-related hormonal variability in plasma metabolome and gut microbiota. No prior procedures were performed on these animals before the start of this study. The mice were kept under specific-pathogen-free conditions with free access to water and food. Mice were housed in groups of 6 per cage in individually ventilated cages (IVCs) with corncob bedding. Environmental conditions were maintained at a temperature of 22~26 °C with about 60% relative humidity and a 12 h light/dark cycle. Environmental enrichment was provided in the form of red plastic house toys. After a 7-day acclimation period, the mice were randomly divided into three groups (water, coffee and high-fat coffee groups, *n* = 6) and administered 200 μL of liquid by oral gavage daily for 14 days at the same time each day. Plasma and fecal samples were collected pre-and post-intervention for metabolomic profiling and 16S rRNA gene sequencing, respectively. The exact plasma and fecal samples collected are specified in Tables S1 and S2.

The black coffee was prepared by mixing instant coffee powder (Luckin Coffee Energizing Bomb Series 03 Intensified Americano, purchased from Luckin Coffee Technology (Hainan) Co., Ltd., Haikou, China) with water at a 1:6 ratio (*w*/*w*). The high-fat coffee consisted of the same coffee powder, water, and light cream (Nestlé All-Purpose Cream, 250 mL, purchased from Nestlé Qingdao Ltd., Qingdao, China) in a 3:5:13 ratio (*w*/*w*/*v*). See Supplementary Material for more details about the chemical composition of the coffee powder and light cream. The coffee concentration was selected based on a previous study [9]. In the coffee group, mice received 0.2 mL of coffee solution daily via gavage, containing 1.98 mg of caffeine. The human equivalent dose (HED), calculated using body surface area normalization [10], was approximately 8.03 mg/kg per day. In the high-fat group, mice received 1.98 mg caffeine and 50 mg of milk fat, corresponding to a fat intake of approximately 12 g/day for a 60 kg adult [11]. All experimental procedures involving animals were approved by the Institutional Animal Care and Use Committee of Peking University Health Science Center.

### 2.2. Metabolomics

Mice were anesthetized using a small-animal anesthesia system RWD R500 (RWD Life Science Co. Ltd., Shenzhen, China), with oxygen flow (typically 0.3~0.5 L/min) and anesthetic concentration (generally 3%~5% for induction) adjusted according to body weight. Blood was collected via retro-orbital plexus bleeding. After centrifugation, a minimum of 100 μL of undiluted plasma was obtained per mouse.

Mouse plasma samples were processed as follows: 100 μL of plasma was mixed with 300 μL of extraction solution (acetonitrile/methanol = 1:1, *v*/*v*), vortexed, and incubated at −20 °C for 30 min for protein precipitation. After centrifugation (13,000× *g*, 4 °C, 15 min), the supernatant was collected, dried under nitrogen, reconstituted, and centrifuged again. The final supernatant was transferred to LC-MS vials.

The LC-MS/MS analysis of samples was conducted on a UHPLC-Orbitrap Exploris 480 system equipped with an ACQUITY HSS T3 column (100 mm × 2.1 mm i.d., 1.8 μm; Waters, Milford, MA, USA). The electrospray ionization (ESI) source operated in both positive and negative modes, with data acquired in data-dependent acquisition (DDA) mode.

The raw data were processed by Progenesis QI v3.0 (Waters Corporation, Milford, MA, USA) software, with metabolites tentatively identified by HMDB (http://www.hmdb.ca/ (accessed on 12 June 2026)), Metlin (https://metlin.scripps.edu/ (accessed on 12 June 2026)) and the self-compiled Majorbio Database (MJDB) of Majorbio Biotechnology Co., Ltd. (Shanghai, China).

See the Supplementary Material for a more detailed metabolomics workflow.

### 2.3. Microbiomics

Total genomic DNA of the microbial community was extracted from fecal samples of C57 mice using a FastPure Stool DNA Isolation Kit (MJYH, Shanghai, China). The extraction procedure was performed strictly following the manufacturer’s protocol, encompassing lysis, centrifugation-based purification, and elution steps. DNA integrity was verified by 1% agarose gel electrophoresis, while its concentration and purity were quantified using a NanoDrop2000 spectrophotometer (Thermo Scientific, Waltham, MA, USA).

The extracted DNA was used as a template to amplify the hypervariable V3–V4 region of the 16S rRNA gene with barcode-indexed primers 338F (5′-ACTCCTACGGGAGGCAGCAG-3′) and 806R (5′-GGACTACHVGGGTWTCTAAT-3′). PCR products were purified and recovered by 2% agarose gel electrophoresis, followed by library construction using a NEXTFLEX Rapid DNA-Seq Kit (Bioo Scientific, Austin, TX, USA). Libraries were quantified, normalized, and subjected to paired-end 250 bp sequencing (PE250) on the Illumina NextSeq 2000 platform (Shanghai Majorbio Bio-pharm Technology Co., Ltd., Shanghai, China).

Paired-end raw sequencing reads were processed using fastp for quality control, FLASH for assembly, and UPARSE v7.1 for chimera removal. Taxonomic annotation of OTUs was performed by aligning sequences against the Silva 16S rRNA gene database (v138) with the RDP classifier (http://rdp.cme.msu.edu/ (accessed on 9 June 2025), version 2.11) at a 70% confidence threshold. Community composition statistics were subsequently generated for each sample across multiple taxonomic levels. For a more detailed description, see the Supplementary Material.

### 2.4. Statistical Analysis

In univariate statistical analysis, normality was assessed using the Shapiro–Wilk test, and homogeneity of variances was evaluated using Levene’s test for each metabolite. An appropriate statistical test (Student’s *t*-test, Welch’s *t*-test or Wilcoxon rank-sum test) was selected for each metabolite based on the assumption checks. A total of 1287 metabolites were tested. To correct for multiple comparisons, the Benjamini–Hochberg false discovery rate (FDR) method was applied to all *p*-values obtained from the group comparisons. Partial Least Squares Discriminant Analysis (PLS-DA) was carried out using the ropls R package (version 1.34.0), and validation was performed using a permutation test (*n* = 200). The microbial and association analysis was performed using the Majorbio platform (https://v.majorbio.com/ (accessed on 12 June 2026)). A chord diagram was constructed using the circlize package (version 0.4.15), and only correlations with absolute coefficient > 0.6 and adjusted *p*-value < 0.05 were included.

## 3. Results and Discussion

### 3.1. Strategy and Characterization of Intervention Mice

**Figure 1:**
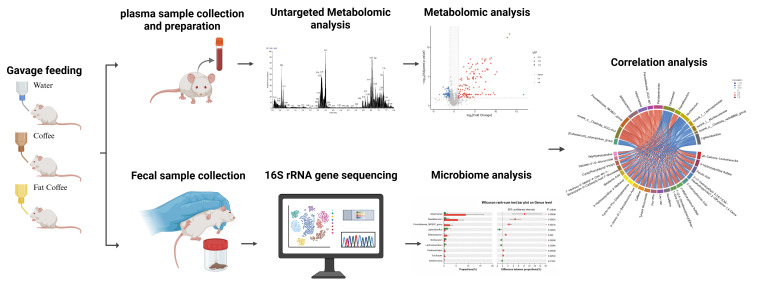
Experimental design and multi-omics analysis workflow. Eighteen mice were randomly assigned to three groups (*n* = 6): water, black coffee, or high-fat coffee, administered by oral gavage for 14 days. Fecal samples were subjected to 16S rRNA sequencing and plasma samples to metabolomic profiling. The two datasets were analyzed independently and then integrated via correlation network analysis to link differential metabolites and microbial genera. * *p* < 0.05, ** *p* < 0.01.

Our strategy is illustrated in Figure 1. Briefly, 18 mice were randomly assigned into three groups (*n* = 6 per group): receiving water, black coffee, or high-fat coffee via oral gavage. Following an intervention of 14 days, fecal and plasma samples were analyzed for 16S rRNA gene sequencing and metabolomic profiling, respectively. These two omics datasets were first analyzed independently to characterize global metabolic and microbial alterations, and then correlation analysis was performed to establish association networks between differential microbial species and metabolites.

We monitored the body weight of the 18 experimental mice at three time points (Day 1 before intervention, Day 7, Day 14) to assess the general physiological impact of the interventions, see Table S3. Statistical analysis using the Kruskal–Wallis nonparametric test revealed no significant differences (*p* > 0.05) in body weight among the three groups across all time points, indicating that neither coffee nor high-fat coffee administration induced overt changes in overall growth. These findings suggest that the following observed metabolic and microbial alterations are unlikely to be confounded by differences in body weight, thereby providing a stable physiological basis for subsequent multi-omics analyses.

### 3.2. Metabolomic Analysis

**Figure 2:**
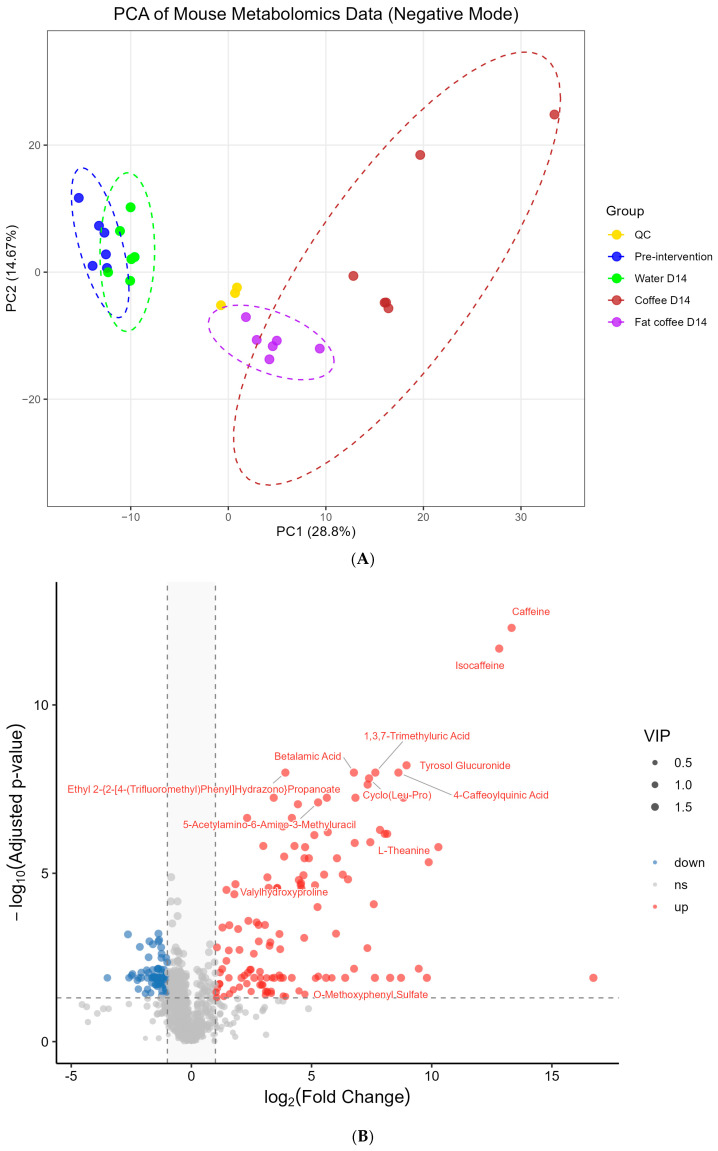

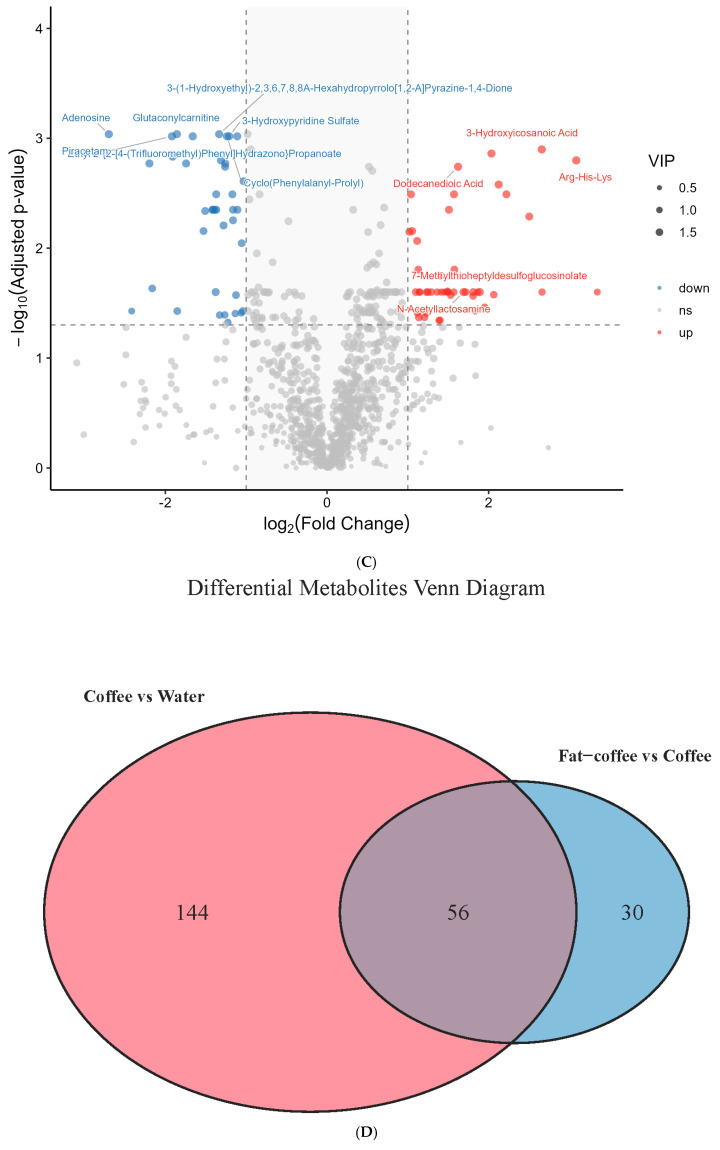
(**A**) PCA score plots of molecular features in negative ion mode. Samples from water group and before intervention group are located on left side, while coffee and high-fat coffee groups were predominantly distributed in right region. Dashed ellipses denote 95% confidence intervals for each group. (**B**) Volcano plot for coffee group compared with water. Significantly altered metabolites defined by |log_2_FC| > 1, *p* adj < 0.05, and VIP > 1. Red: upregulated (higher in coffee); blue: downregulated; grey: not significant. Point size corresponds to VIP value. (**C**) Volcano plot for high-fat coffee group compared with coffee group. Same thresholds and color scheme as (**B**), highlighting metabolic changes specifically induced by lipid enrichment. (**D**) Intersection analysis of significantly dysregulated metabolite; 56 overlapping metabolites represent a core group of metabolic features.

Untargeted HPLC-MS analysis yielded 2218 metabolites successfully tentatively identified through public databases, including HMDB and Lipid Maps, as well as an in-house library from Majorbio. After data pretreatment (see Supplementary Material for details), 1287 stable and reliable metabolites were retained. As shown in Figure 2A, quality control (QC) samples were highly overlapped near the middle of the principal component analysis (PCA) score plot, indicating the sound analytical stability and reproducibility of the metabolomic platform. Samples from the water-treated group clustered closely with those collected at baseline (before intervention), as expected, both localizing to the left side of the PCA plot, suggesting minimal metabolic perturbation in the absence of intervention. In contrast, the coffee and high-fat coffee groups were clearly separated from the water and baseline samples, predominantly distributed in the right region of the PCA space. This distinct shift indicates that both coffee and lipid-enriched coffee induced considerable alterations in the plasma metabolome, which were further supported by subsequent statistical analyses (*t*-test and PLS-DA). Furthermore, a certain degree of separation between the coffee and high-fat coffee groups was observed, implying that lipid enrichment introduced additional metabolic divergence. In line with PCA, the sample correlation plot (Figure S1) also revealed the profound disturbance by coffee and high-fat coffee gavage.

With our focus on two key comparisons—coffee versus water and high-fat coffee versus coffee after the intervention—we performed univariate analysis and PLS-DA to identify differential metabolites. For each metabolite, normality and variance homogeneity were assessed to guide the choice of Student’s *t*-test, Welch’s *t*-test, or Wilcoxon rank-sum test. The PLS-DA permutation test plots are shown in Figure S2A (coffee vs. water) and Figure S2B (high-fat coffee vs. coffee), and no obvious overfitting was observed. As illustrated in the volcano plot in Figure 2B, a substantial number of metabolites were significantly altered in the coffee group compared with the water group, with 126 upregulated and 74 downregulated, based on the following criteria: adjusted *p* < 0.05, fold change (FC) > 2 or < 0.5, and PLS-DA variable importance in projection (VIP) > 1. The most significantly changed metabolites was caffeine, as expected. In parallel, the comparison between the high-fat coffee and coffee groups (Figure 2C) identified 86 significantly altered metabolites, of which 47 were upregulated and 39 were downregulated, suggesting that lipid supplementation altered metabolite abundance in both directions, with a slight predominance of upregulation. Intersection analysis (Figure 2D) of the significant changed metabolites (Figure 2B,C) revealed a set of 56 overlapping metabolites, representing a core group of metabolic features. Notably, all of these shared metabolites exhibited opposite trends between the two comparisons. Specifically, metabolites that were upregulated in the coffee group relative to the water group tended to be downregulated in the high-fat coffee group relative to the coffee group and vice versa.

### 3.3. Microbiomics

**Figure 3:**
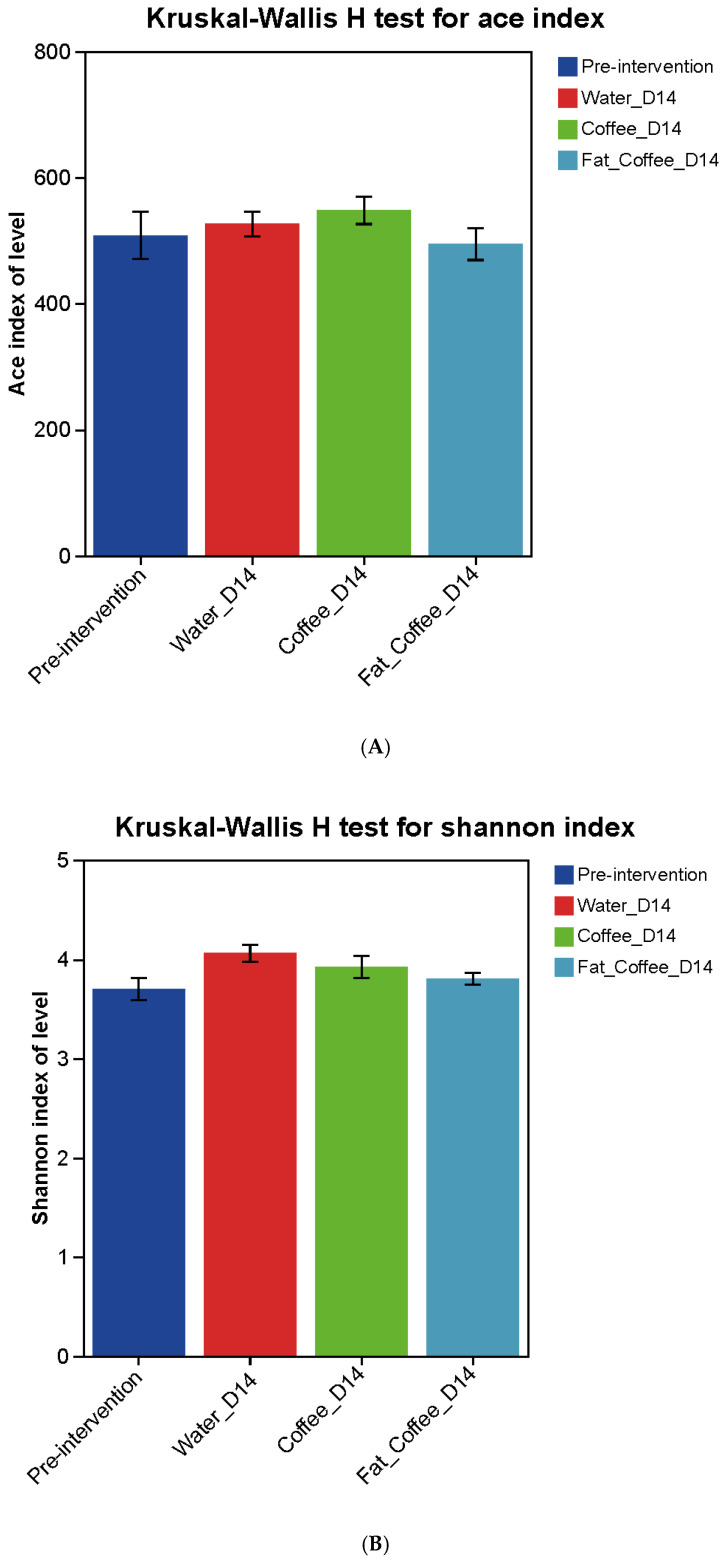

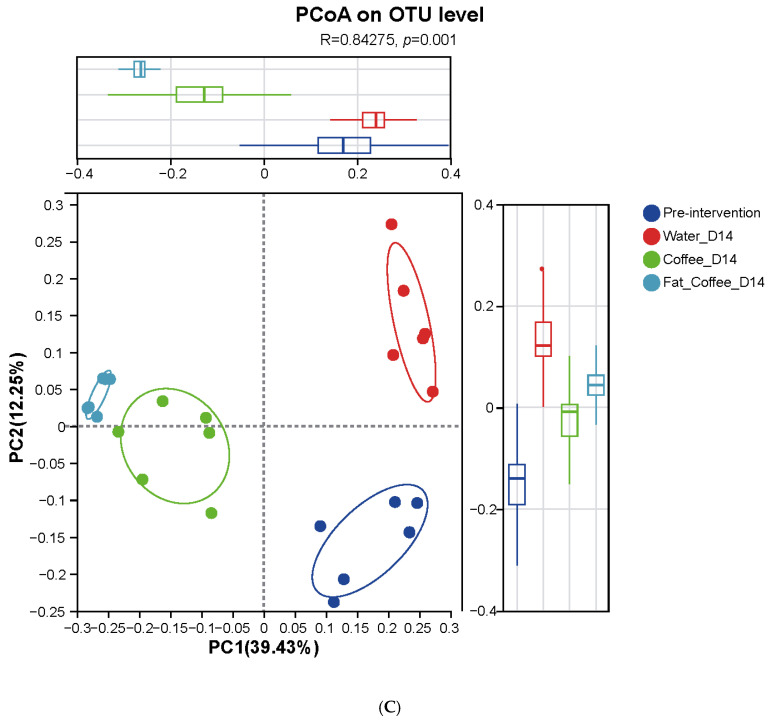
Alpha and beta diversity analysis of the gut microbiota in gavaged mice. Bar plots of (**A**) ACE index and (**B**) Shannon evenness index values across groups. Data are presented as mean ± SD (*n* = 6 mice per group). Statistical significance was assessed using Kruskal–Wallis test followed by FDR correction for multiple comparisons. Significance threshold was set at adjusted *p* < 0.05. (**C**) PCoA plot of microbiota composition for all groups based on Bray–Curtis distance. Each point represents individual mouse sample. Statistical significance of group separation was assessed by ANOSIM.

As illustrated in Figure S3, the rarefaction curves gradually reach a plateau as the sequencing depth increases, indicating adequate sequencing depth for downstream analysis. To evaluate the impact of coffee and high-fat coffee on gut microbial diversity, alpha diversity was first assessed using two commonly applied indices, namely, ACE (species richness) and Shannon (evenness of species distribution). As shown in Figure 3A, B, no significant differences were observed in either the ACE or Shannon indices among the experimental groups based on the Kruskal–Wallis nonparametric test. These results suggest that neither coffee nor high-fat coffee intervention markedly altered the overall richness or diversity of the gut microbiota under the present experimental conditions.

In addition to alpha diversity, beta diversity analysis was conducted to examine differences in microbial community structure across groups. Distance-metrics-based principal coordinates analysis (PCoA) reveals a clear separation pattern among the groups (Figure 3C). Specifically, the samples from the baseline and water-treated groups were primarily distributed on the right side of the PCoA plot, whereas the samples from the coffee and high-fat coffee groups clustered on the left side. Notably, consistent with the PCA results in Figure 2A, a certain degree of separation was also observed between the coffee and high-fat coffee groups, indicating that lipid supplementation further influenced microbial community composition. These findings collectively suggest that both metabolic and microbial profiles were distinctly altered by coffee and its lipid-enriched variant.

**Figure 4:**
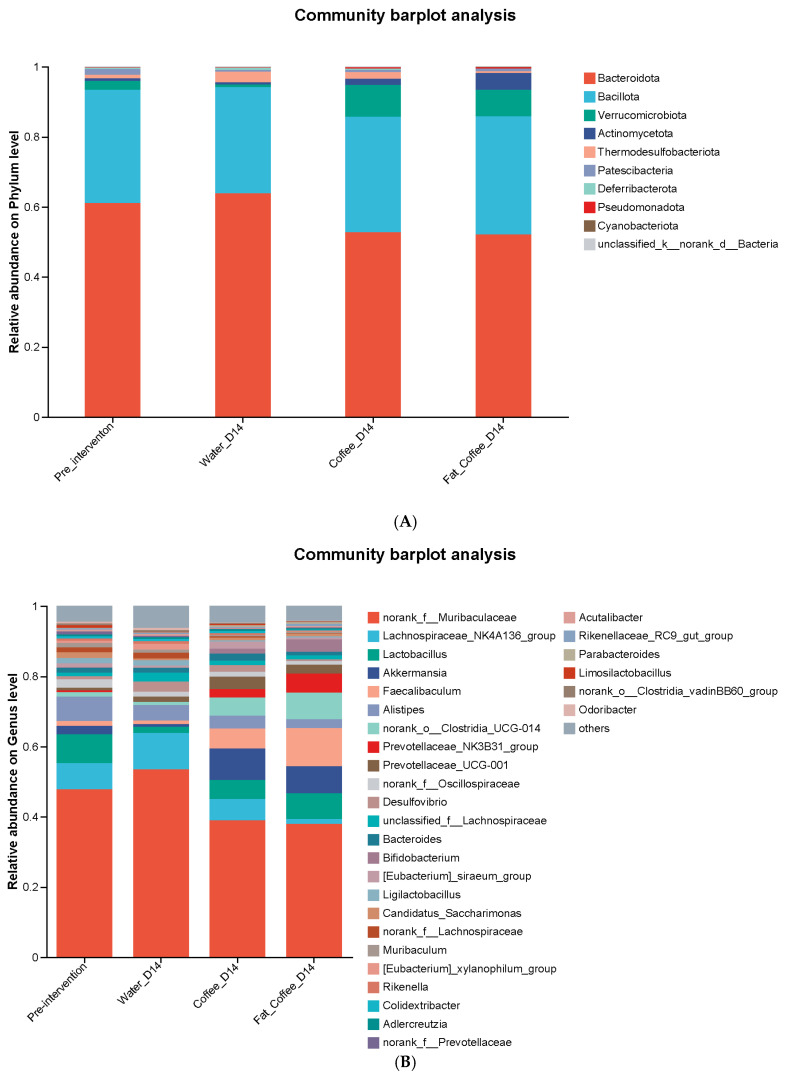

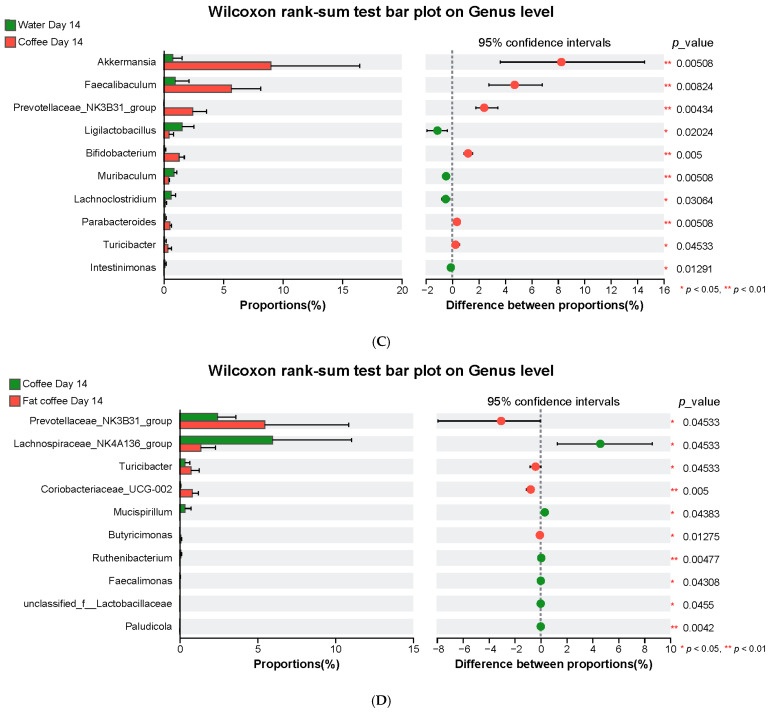
Structure of bacterial communities of gut microbiota at phylum (**A**) and genus (**B**) levels in gavaged mice. (**C**) Wilcoxon rank-sum test bar plot between coffee and water groups on genus level. (**D**) Wilcoxon rank-sum test bar plot between high-fat coffee and coffee groups on genus level. Data are presented as mean ± SD (*n* = 6 mice per group). Statistical significance was assessed using Wilcoxon rank-sum test with FDR correction for multiple comparisons. Significance threshold was set at *p* < 0.05.

The structure of the bacterial communities of the gut microbiota at the phylum and genus levels in gavaged mice is shown in Figure 4A,B respectively. The Wilcoxon rank-sum test between the coffee and water groups revealed a significant increase in the relative abundances of *Akkermansia*, *Bifidobacterium* and *Faecalibaculum*, accompanied by a decrease in *Ligilactobacillus* and *Muribaculum* in coffee group (Figure 4C). These genera have been implicated in host metabolic regulation [12], suggesting that coffee consumption promotes beneficial microbial shifts. In contrast, compared to the coffee group, Coriobacteriaceae UCG-002, *Turicibacter*, and Prevotellaceae NK3B31 group were significantly enriched in the high-fat coffee group, whereas Lachnospiraceae NK4A136 group, *Mucispirillum* and unclassified Lactobacillaceae were reduced (Figure 4D). These findings indicate that the addition of dietary lipids further reshaped the gut microbial community beyond the effects of coffee alone, potentially leading to altered host–microbiota interactions.

### 3.4. Association Analysis Between Metabolome and Microbiome

**Figure 5:**
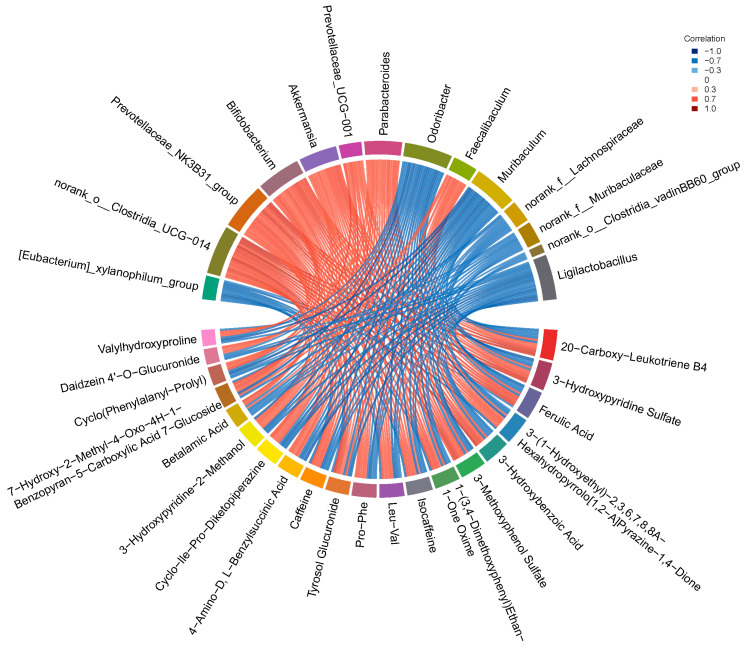
Chord diagram shows correlation between key metabolites and gut microbial genera. Red and blue ribbons represent positive and negative correlations, respectively.

To investigate the potential metabolic interactions between host metabolism and gut microbiota under different dietary interventions, we performed a correlation-based integrative analysis using the 56 overlapping metabolites in Figure 2D and gut microbial genera. Only strong correlations (Benjamini–Hochberg adjusted *p*-value < 0.05 and absolute coefficient > 0.6) were considered. After excluding compounds with ambiguous or unreliable annotations, such as drug-related molecules or xenobiotics, the top 20 metabolites with the highest degrees of connection were selected for visualization using a chord diagram (Figure 5). See Tables S4 and S5 for the complete correlation matrix and regulation details of the top 20 key metabolites, respectively. Strikingly, the majority of these 20 hub metabolites were identified as coffee-specific alkaloids, xanthines, phenolic acids and conjugates. All of these metabolites were upregulated in the coffee group compared to the water group but downregulated in the high-fat coffee group compared to the coffee group. In coffee group, 3-hydroxypyridine sulfate was upregulated, which is in line with previous studies [13,14]. A serum metabolomic study in humans showed 3-hydroxypyridine sulfate positively correlated with coffee consumption after Bonferroni correction for multiple testing [13]. A very recent study reported a strong positive association of 3-hydroxypyridine sulfate with the frequency of coffee intake in premenopausal women [14]. In addition, Cyclo-Ile-Pro-Diketopiperazine and Cyclo(Phenylalanyl-Prolyl) were significantly in increased in the coffee group in our study, which was supported by prior findings that instant coffees differed from all coffee brews in high contents of diketopiperazines [15].

Regarding the microbial counterparts, a set of genera exhibited positive correlations with the majority of these 20 metabolites, including *Bifidobacterium*, *Akkermansia*, *Parabacteroides* and *Faecalibaculum*, all of which are beneficial bacteria. Conversely, genera such as *Odoribacter*, *Ligilactobacillus* and *Muribaculum* showed negative correlations. This observation suggests that a high-fat diet influences host absorption of specific coffee-derived metabolites, potentially alongside changes in the gut microbial community.

### 3.5. Integrated Pathway Analysis

**Figure 6:**
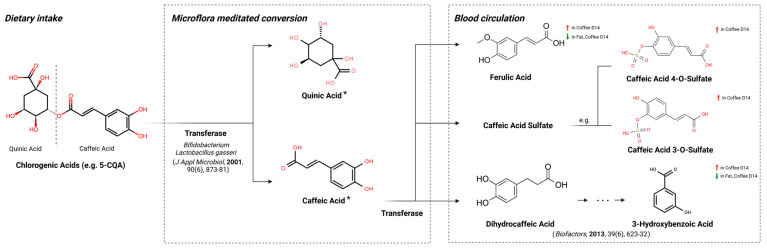
Chlorogenic acid metabolic pathway. Chlorogenic acids are hydrolyzed to quinic acid and caffeic acid via *Bifidobacterium* [23]. Caffeic acid undergoes conversion to ferulic acid, caffeic acid sulfate and dihydrocaffeic acid, which are further degraded by gut bacteria to 3-hydroxybenzoic acid [21]. Arrows indicate significant changes (red ↑ = increased, green ↓ = decreased, adjusted *p* < 0.05). Asterisks * indicate metabolites detected but not significantly changed.

Among the 20 key metabolites obtained in the association analysis, two compounds, ferulic acid and 3-hydroxybenzoic acid, are involved in the chlorogenic acid metabolic pathway (Figure 6) [16]. Chlorogenic acids, a group of compounds comprising hydroxycinnamates linked to quinic acid, are essential substances in coffee [17]. They are reported to be the main antioxidants in coffee and to contribute to the health benefits associated with coffee consumption [18,19]. In vivo, chlorogenic acids are hydrolyzed into quinic acid and caffeic acid. The latter can be further metabolized along three routes: (i) sulfation to caffeic acid 3-O-sulfate and caffeic acid 4-O-sulfate [17], (ii) COMT-mediated methylation to ferulic acid [20], and (iii) gut bacterial degradation into simpler compounds such as 3-hydroxybenzoic acid [21]. The bioavailability of chlorogenic acid is highly dependent on the catabolism of the gut microbiota. Approximately one-third of chlorogenic acid is absorbed in the stomach and small intestine, while the remaining two-thirds enter the large intestine [22]. Certain gut microbial taxa, particularly members of the genera *Bifidobacterium* and *Lactobacillus* (e.g., *Bifidobacterium lactis* and *Lactobacillus gasseri*), possess enzymatic activities capable of hydrolyzing chlorogenic acids, thereby facilitating the production of absorbable phenolic compounds in the large intestine [23,24].

Our metabolomic results showed that, compared with the water group, the coffee-treated mice exhibited significantly elevated levels of ferulic acid and 3-hydroxybenzoic acid. In contrast, these metabolites were significantly reduced in the high-fat coffee group relative to the coffee group. The tandem MS spectrum of ferulic acid and 3-hydroxybenzoic acid are provided in Figure S4, which is in good agreement with the literature [25,26]. Microbiome analysis revealed a significant enrichment of *Bifidobacterium* in the coffee group, whereas a marked reduction in Lactobacillaceae was observed in the high-fat coffee group (Figure 4C,D). These results suggest that the increased levels of caffeic-acid-related metabolites in the coffee group were associated with the presence of lipase-active microbial taxa, such as *Bifidobacterium*, which facilitate the breakdown of chlorogenic acids. Conversely, the decreased abundance of Lactobacillaceae in the high-fat coffee group may have contributed to the reduced production of these metabolites, potentially through the suppression of microbial metabolic activity. It is noteworthy that caffeic acid 3-O-sulfate and caffeic acid 4-O-sulfate were also significantly enriched in the coffee group (vs. water group), while both metabolites showed a downward trend in the high-fat coffee group (vs. the coffee group), with raw *p*-values of 0.046 and 0.037 and log2 fold changes of −0.036 and −0.040, respectively. Note that ferulic acid and 3-hydroxybenzoic acid are not bacterially synthesized de novo but are degradation products derived from coffee chlorogenic acids via microbial metabolism. Quinic acid and caffeic acid were also detected in our data, although their levels did not change significantly. Chlorogenic acids (e.g., 5-O-caffeoylquinic acid) were not directly detected in our experiment. This aligns with previous reports that they are rapidly absorbed and extensively metabolized, resulting in limited systemic presence [27]. Taken together, these findings suggest a link between lipid-induced gut microbiota alterations and the reduced conversion of chlorogenic acids into bioactive metabolites such as ferulic acid and 3-hydroxybenzoic acid, which are associated with coffee’s anti-inflammatory and antioxidant properties.

## 4. Limitations

Although our high-fat coffee versus coffee comparison controlled for the presence of coffee, the lack of a fat-only group precludes us from distinguishing whether the observed changes are driven by the lipid component or by its interaction with coffee. While MSI level 2 annotation provides strong structural evidence, future targeted analyses using authentic standards are warranted to confirm their absolute levels.

## 5. Conclusions

In summary, this study provides a comprehensive multi-omics characterization of the effects of coffee and high-fat coffee on host metabolism and gut microbiota in a mouse model. Coffee consumption induces significant alterations in the plasma metabolome and gut microbial composition, while the addition of dietary lipids reshapes these responses, representing a partial reversal of the coffee-induced effects, particularly at the metabolome level. Association and pathway analyses identified the chlorogenic acid pathway as a key axis underlying the differential effects of the interventions. Coffee intake promoted the accumulation of bioactive metabolites, such as ferulic acid, 3-hydroxybenzoic acid and caffeic acid sulfates, which are known for their anti-inflammatory and antioxidant properties. However, these beneficial metabolites were markedly reduced in the high-fat coffee group. These exploratory findings suggest that lipid enrichment modifies coffee-associated plasma metabolite and fecal microbiota profiles, including potential effects on chlorogenic-acid-related metabolites and taxa such as *Bifidobacterium* and Lactobacillaceae; however, mechanistic and functional validation is required. This study advances our understanding of diet–microbiota–metabolite interactions and underscores the importance of considering beverage composition in nutritional and metabolic research. Future ex vivo anaerobic culture studies with coffee or high-fat coffee are needed to elucidate whether dietary lipids suppress microbial metabolism directly or via the gut environment. Functional phenotypic parameters such as blood glucose and oxidative stress markers are also necessary to validate the biological implications suggested by our -omics data.

## Data Availability

Metabolomic data are available via the OMIX database at the China National Center for Bioinformation/Beijing Institute of Genomics, Chinese Academy of Sciences (https://ngdc.cncb.ac.cn/omix (accessed on 12 June 2026)) under accession number OMIX016182; 16S rRNA sequencing data are available on NCBI (https://www.ncbi.nlm.nih.gov (accessed on 12 June 2026)) under accession number PRJNA1452546.

## Supplementary Materials

The following supporting information can be downloaded at https://www.mdpi.com/article/10.3390/nu18121939/s1, Metabolomic analysis: detailed workflow of metabolomics; microbiomics: detailed workflow of 16S rRNA sequencing; Table S1: Plasma for metabolomics; Table S2: Fecal for microbiome analyses; Table S3: Body weight (g) of mice at different time points; Table S4: Correlation matrix of hub metabolites and gut microbial genera; Table S5: Summary of top 20 key metabolites; Figure S1: Sample correlation heatmap showing pairwise Pearson correlation coefficients among all samples; Figure S2: PLS-DA permutation test (*n* = 200) for (A) coffee vs. water and (B) fat-coffee vs. coffee. No overfitting was observed; Figure S3: Rarefaction curve of the 16S rRNA sequencing data; Figure S4: MS/MS spectra of (A) ferulic acid (C_10_H_10_O_4_) and (B) 3-hydroxybenzoic acid (C_7_H_6_O_3_).
