# Supplementary material for: Integrated Metabolomic and Microbiome Profiling Reveals Divergent Effects of No- and High-Fat Coffee in Mice
Source: Nutrients. 2026 Jun 16;18(12):1939. doi: 10.3390/nu18121939 (PMC13305275; doi:10.3390/nu18121939)
Supplement: Supplementary file 1 [file nutrients-18-01939-s001.zip › Supplementary material.pdf]

Integrative Metabolomic and Microbiome Profiling Reveals Divergent Effects of Coffee and High-Fat Coffee in Mice

Xinye Gong[1]†, Yuelin Wang[1]†, Wenbo Chu[1], Yijie Zhao[2], Jia Liu[1]*, Qinghua Zou[1]*

[1] Department of Microbiology, School of Basic Medical Sciences, Peking University Health Science Center, Beijing, 100191, China

[2] Department of Laboratory Animal Science, Peking University Health Science Center, Beijing, 100191, China

*** Corresponding author contact information**

Jia Liu, Ph.D.

E-mail: liujia894@bjmu.edu.cn

Qinghua Zou, Ph.D.

E-mail: zouqinghua@bjmu.edu.cn

† Xinye Gong and Yuelin Wang contributed equally to this work.

Preparation of Coffee and High-Fat Coffee Solutions

The coffee solution was prepared by dissolving 6 g of instant coffee powder (Luckin Coffee Energizing Bomb Series 03 Intensified Americano, purchased from Luckin Coffee Technology (Hainan) Co., Ltd.) in 36 mL of water. According to the manufacturer's information, the primary components per 2.3g of powder were: caffeine138.34 mg, energy 32 kJ, protein 0.7 g, fat 0 g, carbohydrate 1.2 g, sugars 0 g, sodium 2 mg. The high-fat coffee solution was prepared by dissolving 6 g of instant coffee powder (Luckin Coffee Energizing Bomb Series 03 Intensified Americano, purchased from Luckin Coffee Technology (Hainan) Co., Ltd.) in 10 mL of water, and then adding 26 mL of commercial light cream (Nestlé All-Purpose Cream, 250 mL, purchased from Nestlé Qingdao Ltd.) to the above coffee solution. According to the manufacturer's information, the nutritional composition of the light cream per 100 mL was: energy 1353 kJ, protein 1.2 g, fat 35.0 g, carbohydrate 2.2 g, sodium 30 mg. Ingredient List: Fresh Milk (≥ 70%), Anhydrous Milk Fat, Lecithin, Mono- and Diglycerides of Fatty Acids, Polysorbate 80, Calcium Chloride, Microcrystalline Cellulose, Sodium Carboxymethyl Cellulose, White Sugar.

Metabolomic analysis

Mouse plasma samples were processed as follows: 100 μL plasma was mixed with 300 μL extraction solution (acetonitrile/methanol = 1:1, v/v), vortexed, and incubated at -20 °C for 30 min for protein precipitation. After centrifugation (13,000 × g, 4 °C, 15 min), the supernatant was collected, dried under nitrogen, reconstituted with 100 μL acetonitrile/water (1:1, V/V), and centrifuged again. The final supernatant was transferred to LC-MS vials. As a part of the system conditioning and quality control process, a pooled quality control sample (QC) was prepared by mixing equal volumes of all samples. The QC samples were disposed and tested in the same manner as the analytic samples. QC samples were injected at regular intervals (every 8 samples) to monitor the stability of the analysis.

The LC-MS/MS analysis was conducted on a UHPLC-Orbitrap Exploris 480 system (Thermo Fisher Scientific, Bremen, Germany) equipped with an ACQUITY HSS T3 column (100 mm × 2.1 mm i.d., 1.8 μm; Waters, USA) at Majorbio Bio-Pharm

Technology Co. Ltd. (Shanghai, China). The mobile phase A consisted of 95% water and 5% acetonitrile (containing 0.1% formic acid), and mobile phase B consisted of 47.5% acetonitrile, 47.5% isopropanol, and 5% water (containing 0.1% formic acid). The gradient program was as follows: 0.0% B from 0.0 ~ 3.0 min, increased to 20.0% B at 3.0 min, then to 35.0% B at 4.5 min, followed by a ramp to 100.0% B at 5.0 min, held at 100.0% B until 6.3 min, then returned to 0.0% B at 6.4 min, and re-equilibrated at 0.0% B until 8.0 min. The flow rate was 0.40 mL/min and the column temperature was 40℃. The injection volume was 3 μL.

Mass Spectrometer equipped with an electrospray ionization (ESI) source operating in both positive mode and negative mode. The optimal conditions were set as followed: source temperature at 400 °C; heath gas flow rate at 50 arb; Aux gas flow rate at 15 arb; ion-spray voltage floating (ISVF) at -2800 V in negative mode and 3400 V in positive mode, respectively; Normalized collision energy, 20-40-60V rolling for MS/MS. Data acquisition was performed with the Data Dependent Acquisition (DDA) mode. The detection was carried out over a mass range of 70 ~ 1050 m/z.

Raw LC-MS data were imported into Progenesis QI (Waters Corporation, Milford, MA, USA) for metabolomics data processing including baseline filtering, peak picking, integration, retention time correction, and peak alignment. For compound identification, MS and MS/MS spectral data were matched against public metabolomics databases, including the Human Metabolome Database (HMDB, http://www.hmdb.ca/) and Metlin (https://metlin.scripps.edu/), as well as an in-house library from Majorbio (Shanghai, China). Metabolites were retained only if they had a detection rate of at least 80% in all four experimental groups. After filtering, any remaining missing values were imputed using the k-nearest neighbors (KNN) algorithm. Sum normalization was applied to each sample. QC samples were first evaluated for reproducibility by calculating the relative standard deviation (RSD) of each metabolite across QC injections. Following quality assessment, metabolites with RSD > 20% across QC samples were excluded to retain only analytically reliable features. For univariate analysis (e.g., volcano plots), data were $\log_2$-transformed. For multivariate analysis (e.g., PCA), data were $\log_{10}$-transformed and then standardized using z-score scaling.

Microbiomics

Total genomic DNA of the microbial community was extracted from fecal samples of C57 mice using the FastPure Stool DNA Isolation Kit (MJYH, Shanghai, China). The extraction procedure was performed strictly following the manufacturer's protocol, encompassing lysis, centrifugation-based purification, and elution steps. DNA integrity was verified by 1% agarose gel electrophoresis, while its concentration and purity were quantified using a NanoDrop2000 spectrophotometer (Thermo Scientific, USA).

The extracted DNA was used as a template to amplify the hypervariable V3–V4 region of the 16S rRNA gene with barcode-indexed primers 338F (5'-ACTCCTACGGGAGGCAGCAG-3') and 806R (5'-GGACTACHVGGGTWTCTAAT-3'). PCR products were purified and recovered by 2% agarose gel electrophoresis, followed by library construction using the NEXTFLEX Rapid DNA-Seq Kit (Bioo Scientific, USA). Libraries were quantified, normalized, and subjected to paired-end 250-bp sequencing (PE250) on the Illumina NextSeq 2000 platform (Shanghai Majorbio Bio-pharm Technology Co., Ltd, China).

Raw FASTQ files were de-multiplexed using an in-house perl script, and then quality-filtered by fastp v.0.23.4 and merged by FLASH version 1.2.7 with the following criteria: (i) the reads were truncated at any site receiving an average quality score of < 20 over a 10 bp sliding window, and the truncated reads shorter than 50 bp were discarded, reads containing ambiguous characters were also discarded; (ii) only overlapping sequences longer than 10 bp were assembled according to their overlapped sequence. The maximum mismatch ratio of overlap region is 0.2. Reads that could not be assembled were discarded; (iii) Samples were distinguished according to the barcode and primers, and the sequence direction was adjusted, exact barcode matching, 2 nucleotide mismatch in primer matching. Then the optimized sequences were clustered into operational taxonomic units (OTUs) using USEARCH v.11 with 97% sequence similarity level. Chimeras were detected and removed during OTU clustering using the built-in chimera detection method in USEARCH. The most abundant sequence for each OTU was selected as a representative sequence. To minimize the effects of sequencing depth on alpha and beta diversity measure, the number of 16S rRNA gene sequences

from each sample were rarefied to 20,000, which still yielded an average Good's coverage of 99.09%, respectively. The taxonomy of each OTU representative sequence was analyzed by RDP Classifier v.11.5 against the 16S rRNA gene database (e.g. Silva v138.2) using confidence threshold of 0.7. No sample was excluded.

The metabolomic and microbiome analyses for each individual sample were performed without technical replicates. The n = 6 per group represents biological replicates.

Table S1. Plasma for metabolomics

mice No.	before intervention	gavage	Day 14
1	P1	water	P1_D14
2	P2	water	P2_D14
3	P3	water	P3_D14
4	P4	water	P4_D14
5	P5	water	P5_D14
6	P6	water	P6_D14
7	P7	coffee	P7_D14
8	P8	coffee	P8_D14
9	P9	coffee	P9_D14
10	P10	coffee	P10_D14
11	P11	coffee	P11_D14
12	P12	coffee	P12_D14
13	P13	high-fat coffee	P13_D14
14	P14	high-fat coffee	P14_D14
15	P15	high-fat coffee	P15_D14
16	P16	high-fat coffee	P16_D14
17	P17	high-fat coffee	P17_D14
18	P18	high-fat coffee	P18_D14

The exact plasma samples collected are highlighted.

Table S2. Fecal for microbiome analyses

mice No.	before intervention	gavage	Day 14
1	F1	water	F1_D14
2	F2	water	F2_D14
3	F3	water	F3_D14
4	F4	water	F4_D14
5	F5	water	F5_D14
6	F6	water	F6_D14
7	F7	coffee	F7_D14
8	F8	coffee	F8_D14
9	F9	coffee	F9_D14
10	F10	coffee	F10_D14
11	F11	coffee	F11_D14
12	F12	coffee	F12_D14
13	F13	high-fat coffee	F13_D14
14	F14	high-fat coffee	F14_D14
15	F15	high-fat coffee	F15_D14
16	F16	high-fat coffee	F16_D14
17	F17	high-fat coffee	F17_D14
18	F18	high-fat coffee	F18_D14

The exact fecal samples collected are highlighted.

Table S3. Body weight (g) of mice at different time points.

Group	No.	Day 1	Day 8	Day 14
Water	1	17.78	19.85	20.93
	2	22.6	24.84	26.73
	3	20.13	21.38	22.81
	4	20.84	22.15	23.77
	5	18.42	18.86	19.60
	6	19.08	20.97	21.89
Coffee	7	19.34	20.51	21.57
	8	18.84	21.18	21.24
	9	19.12	20.38	20.28
	10	20.05	22.76	23.02
	11	19.38	19.12	18.97
	12	18.73	20.08	20.27
High-fat Coffee	13	19.50	21.74	23.12
	14	18.50	21.27	21.96
	15	20.41	22.65	23.09
	16	18.24	19.93	20.48
	17	18.38	20.43	20.88
	18	20.17	21.41	22.05

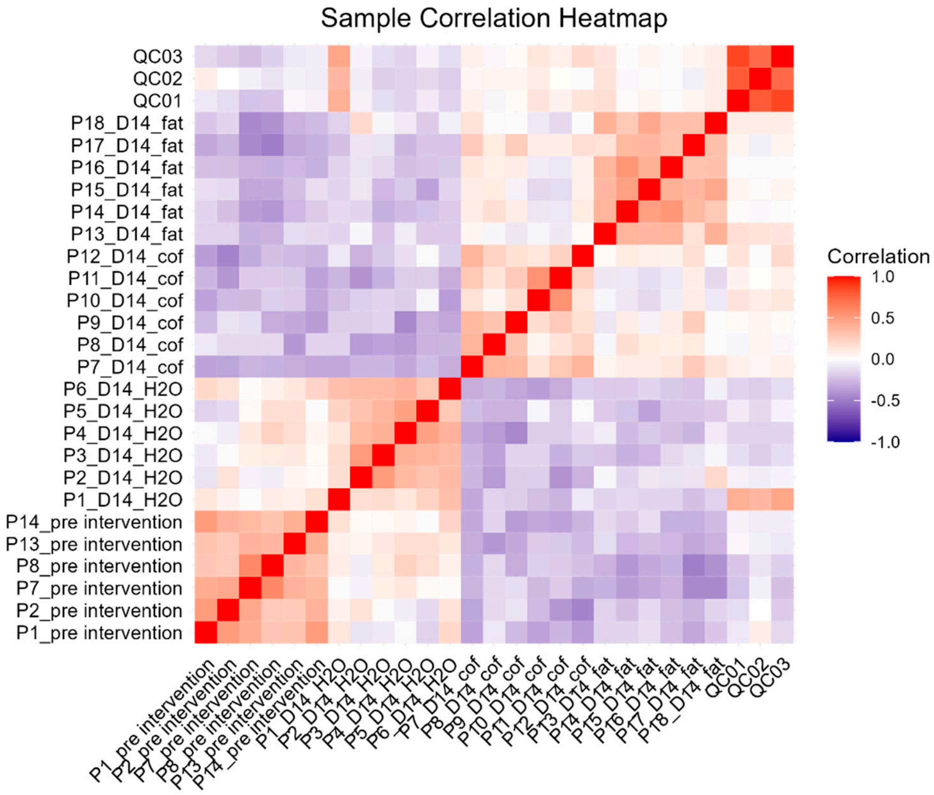

Figure S1. Sample correlation heatmap showing pairwise Pearson correlation coefficients among all samples. Color gradient: red (r = 1, positive correlation), white (r = 0), dark blue (r = –1, negative correlation). This plot assesses sample similarity and relativity prior to downstream analysis.

(A)

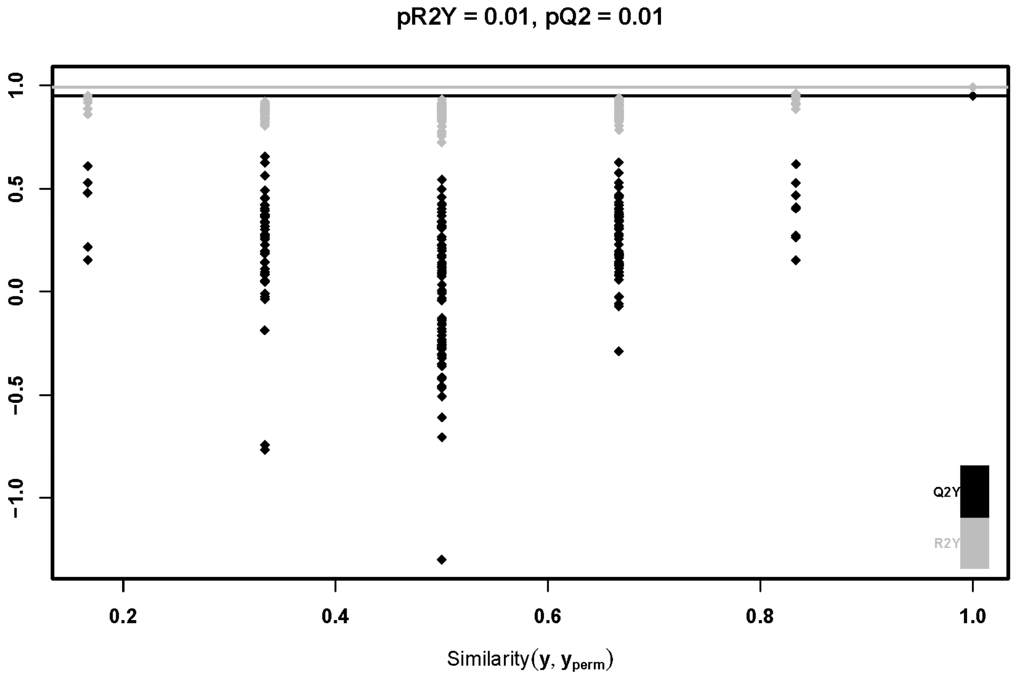

(B)

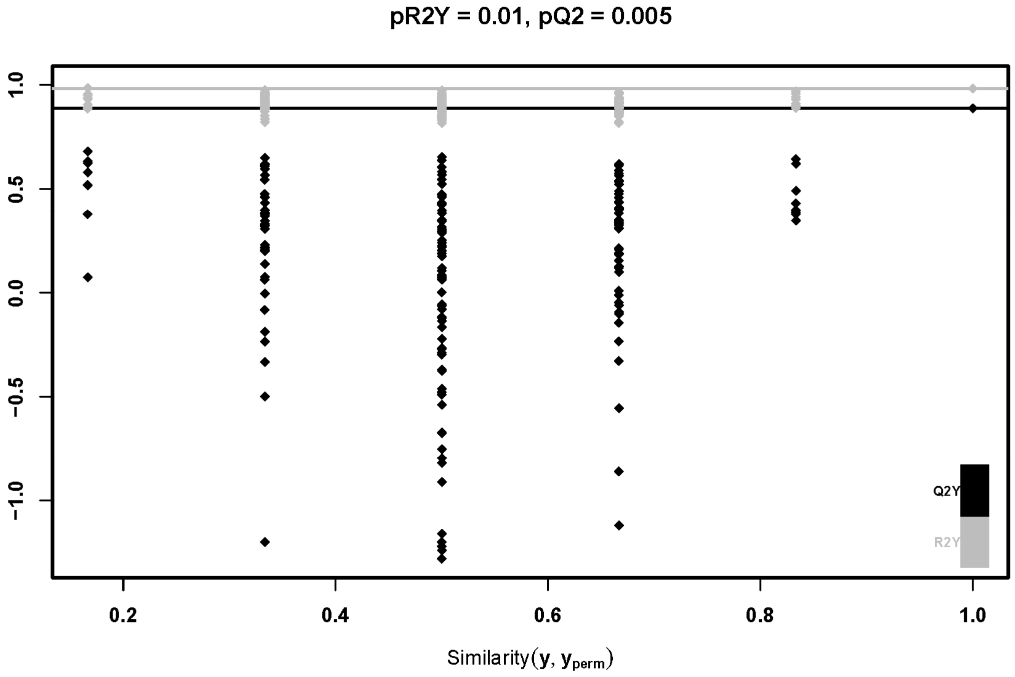

Figure S2. PLS-DA permutation test (n = 200) for (A) coffee vs. water and (B) fat-coffee vs. coffee respectively. No over-fitting was observed.

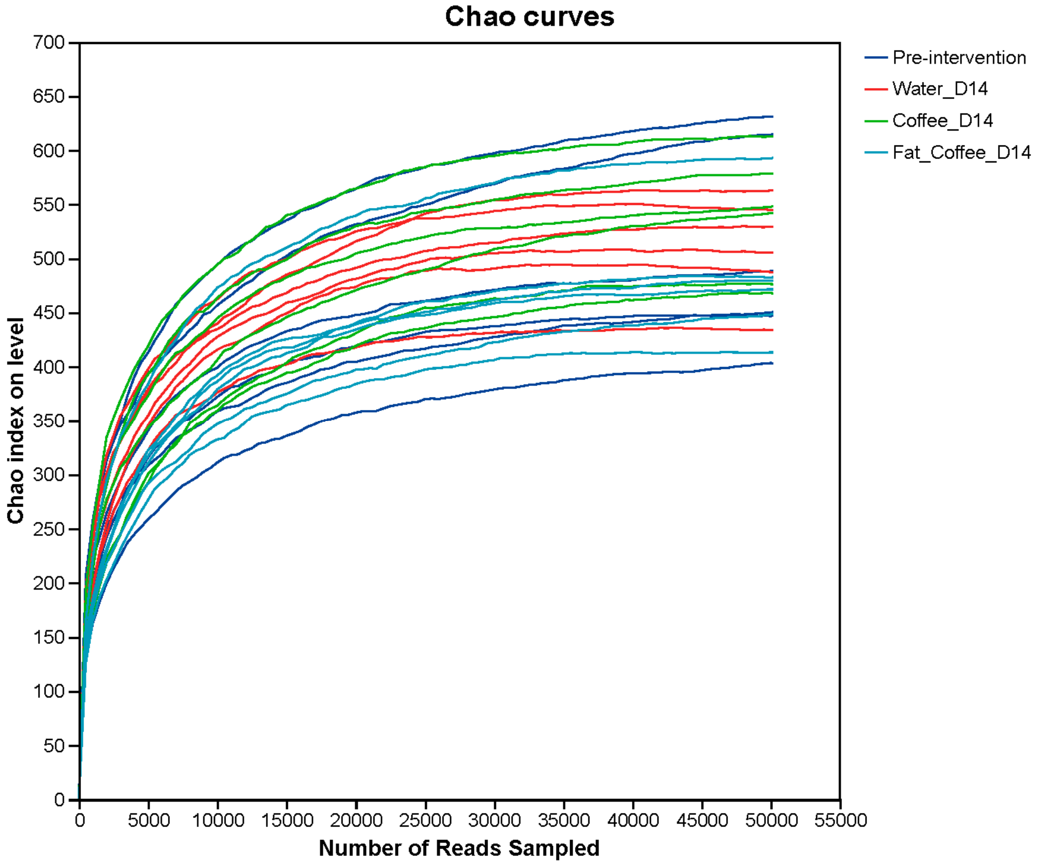

Figure S3. Rarefaction Curve of the 16S rRNA sequencing data.

(A)

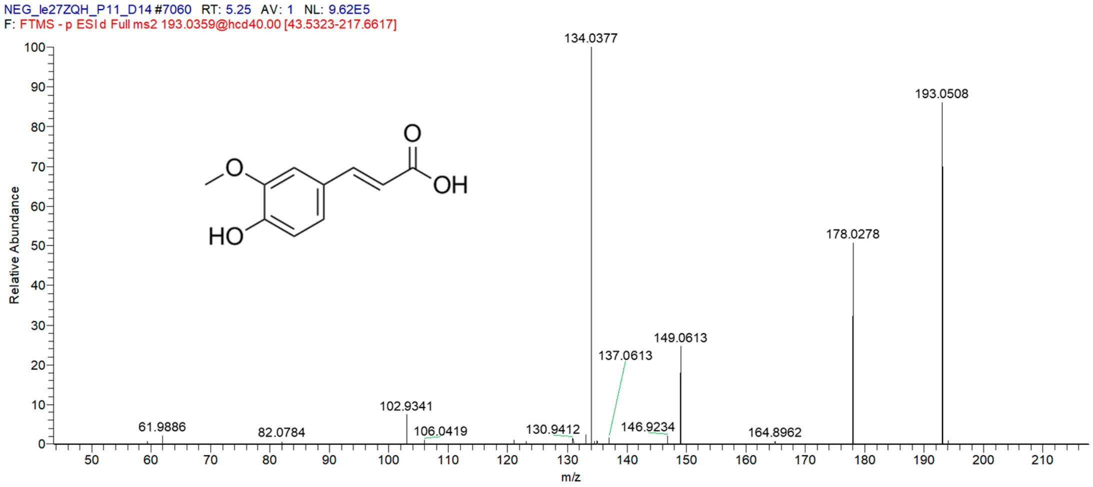

(B)

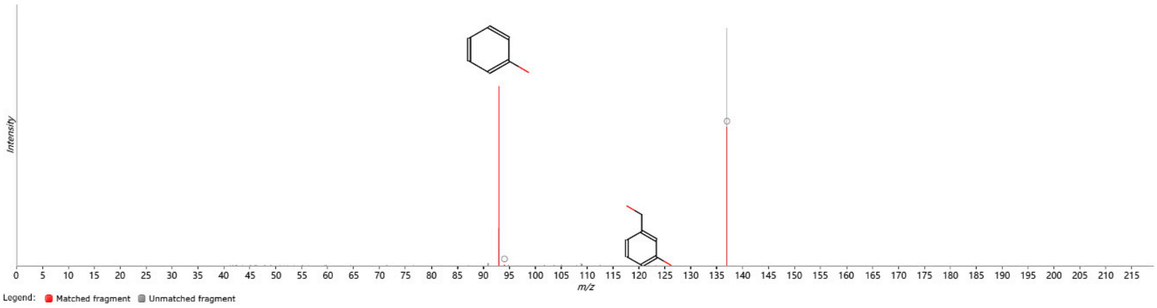

Figure S4. MS/MS spectra of (A) ferulic acid ($C_{10}H_{10}O_4$) and (B) 3-hydroxybenzoic acid ($C_7H_6O_3$).
